# Imaging in severe sepsis and septic shock: is early radiological identification of occult sources of infection needed?

**DOI:** 10.1186/cc14015

**Published:** 2014-12-03

**Authors:** A Creamer, J Keep

**Affiliations:** 1 Emergency Department, Kings College Hospital, London, UK

## Introduction

The importance of imaging in establishing the focus of infection is recognised in current guidelines for the management of severe sepsis [1], with decisions regarding timing and modality of imaging left to the physicians' clinical judgement. In the emergency department (ED), clinical assessment combined with bedside investigations of chest X-ray (CXR) and urine dip can be used to confirm the two most common sources [2]. However, they may fail to identify occult sources of infection, such as intraabdominal collections and abscesses, the treatment of which may require alteration of empirical treatment or be refractory to antibiotic therapy alone. Further imaging is necessary to confirm the focus so that optimal treatment can be achieved.

## Methods

The study cohort was composed of 50 consecutive patients who met the criteria for severe sepsis [1] attending the ED in 2013. Electronic and paper patient records and radiology results were analysed. All radiological studies done in the first 72 hours following attendance were included in the study.

## Results

**Figure 1:**
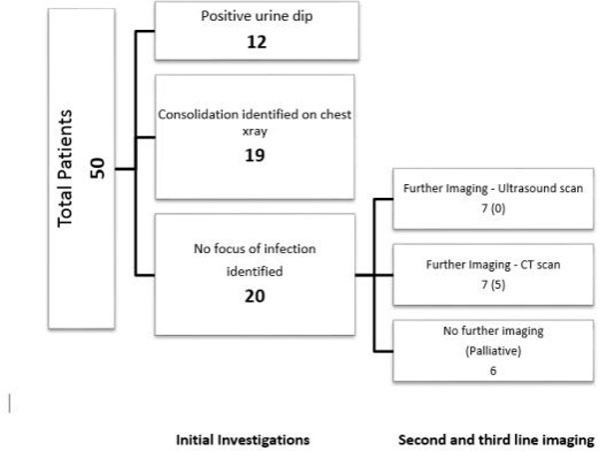
**Initial and further investigations performed (number where a septic focus was identified in parentheses)**.

CXR was performed as the initial investigation in 49 of the 50 patients (98%). The median time from arrival at the ED to initial imaging was 1 hour:00 minutes (range 0 hours:04 minutes to 4 hours:25 minutes). Initial investigations in the ED of CXR and urine dip identified a septic focus in 30 of 50 patients (60%). Fourteen of the remaining 20 went on to have one or more further imaging studies. Figure 1 outlines the second-line and third-line radiological investigations performed (number where a septic focus was identified in parentheses). The median time to first positive imaging was 0 hours:50 minutes (range 0 hours:04 minutes to 40 hours), with the source remaining unidentified by imaging and urine testing in 15 of the 50 patients.

## Conclusion

Our results indicate that simple bedside investigations are able to identify a focus of infection in 60% of patients presenting to the ED with severe sepsis. Our results support the continued use of CXR as the initial imaging modality in severe sepsis, but also demonstrate the benefit of further imaging in confirming the focus of infection and to guide definitive treatment. Instances where further imaging was delayed by several days highlight the need for guidelines detailing which investigations should be done and in what time frame.
